# Fish oil supplementation induces expression of genes related to cell cycle, endoplasmic reticulum stress and apoptosis in peripheral blood mononuclear cells: a transcriptomic approach

**DOI:** 10.1111/joim.12217

**Published:** 2014-03-20

**Authors:** M C W Myhrstad, S M Ulven, C-C Günther, I Ottestad, M Holden, E Ryeng, G I Borge, A Kohler, K W Brønner, M Thoresen, K B Holven

**Affiliations:** 1 Faculty of Health Sciences, Department of Health, Nutrition and Management, Oslo and Akershus University College of Applied SciencesOslo, Norway; 2 Norwegian Computing CenterOslo, Norway; 3 Department of Cancer Research and Molecular Medicine, Norwegian University of Science and TechnologyTrondheim, Norway; 4 Nofima, Norwegian Institute of Food, Fisheries and Aquaculture ResearchÅs, Norway; 5 Department of Mathematical Sciences and Technology, Centre for Integrative Genetics (CIGENE), Norwegian University of Life ScienceÅs, Norway; 6 Centre for Research and Development, TINE SAKalbakken, Oslo, Norway; 7 Department of Biostatistics, Institute for Basic Medical Sciences, University of OsloOslo, Norway; 8 Department of Nutrition, Institute for Basic Medical Sciences, University of OsloOslo, Norway

**Keywords:** fish oil, intervention, n-3 fatty acid, peripheral blood mononuclear cells, transcriptome

## Abstract

**Background:**

Fish oil supplementation has been shown to alter gene expression of mononuclear cells both *in vitro* and *in vivo*. However, little is known about the total transcriptome profile in healthy subjects after intake of fish oil. We therefore investigated the gene expression profile in peripheral blood mononuclear cells (PBMCs) after intake of fish oil for 7 weeks using transcriptome analyses.

**Design:**

In a 7-week, double-blinded, randomized, controlled, parallel-group study, healthy subjects received 8 g day^−1^ fish oil (1.6 g day^−1^ eicosapentaenoic acid + docosahexaenoic acid) (*n *=* *17) or 8 g day^−1^ high oleic sunflower oil (*n *=* *19). Microarray analyses of RNA isolated from PBMCs were performed at baseline and after 7 weeks of intervention.

**Results:**

Cell cycle, DNA packaging and chromosome organization are biological processes found to be upregulated after intake of fish oil compared to high oleic sunflower oil using a moderated *t*-test. In addition, gene set enrichment analysis identified several enriched gene sets after intake of fish oil. The genes contributing to the significantly different gene sets in the subjects given fish oil compared with the control group are involved in cell cycle, endoplasmic reticulum (ER) stress and apoptosis. Gene transcripts with common motifs for 35 known transcription factors including E2F, TP53 and ATF4 were upregulated after intake of fish oil.

**Conclusion:**

We have shown that intake of fish oil for 7 weeks modulates gene expression in PBMCs of healthy subjects. The increased expression of genes related to cell cycle, ER stress and apoptosis suggests that intake of fish oil may modulate basic cellular processes involved in normal cellular function.

## Introduction

It is well documented that consumption of fish and fish oil (FO) rich in the marine n-3 fatty acids eicosapentaenoic acid (EPA, 20:5 n-3) and docosahexaenoic acid (DHA, 22:6 n-3) has a positive health effect in terms of several lifestyle-related diseases such as cardiovascular disease (CVD) 1–3. However, inconsistency of the efficacy of the marine n-3 fatty acids on CVD outcome was recently demonstrated in a meta-analysis including 20 randomized controlled trials 4. Studies exploring the cellular processes specifically modulated by marine n-3 fatty acids *in vivo* are therefore needed.

A low chronic state of inflammation can result in oxidative stress and may therefore promote the development and progression of lifestyle-related diseases 5–7. It has been hypothesized that inflammation is susceptible to nutritional influences and, in particular, n-3 fatty acids have been associated with reduced plasma levels of inflammatory markers in epidemiological studies 8–10. However, the effect seems to be small, and no firm conclusion can be drawn about the influence of marine n-3 fatty acids on circulating inflammatory markers in healthy individuals or those with CVD 11. Other approaches including more sensitive techniques to accurately measure the nutritional influence of these fatty acids on inflammation and other nutrient-regulated metabolic processes are therefore warranted.

The ability of marine n-3 fatty acids to alter gene expression has been clearly demonstrated, and this may account for their potential beneficial health effects. Fatty acids can regulate expression of genes by acting as ligands for the peroxisomal proliferator-activated receptors (PPARs) 12. Activation of PPARs is known to regulate the expression of genes involved in lipid metabolism and inflammation 13. n-3 fatty acids are also known to reduce the activation of the transcription factor nuclear factor kappa B 14 and the Toll-like receptors 15. In addition, it has been shown that signalling pathways upstream of transcription are modulated by n-3 fatty acids. The G protein-coupled receptor GPR120 was recently identified as a receptor/sensor involved in the anti-inflammatory effects of n-3 fatty acids 16.

The peripheral blood mononuclear cells (PBMCs) include monocytes and lymphocytes. PBMCs play a vital role in inflammation and, hence, may be important in inflammatory-related diseases. Both monocytes and lymphocytes are actively involved in the development of atherosclerotic plaques. Leukocytes respond to changes in the plasma lipid levels and systemic inflammation by regulating a network of genes, including those involved in immune response and lipid and fatty acid metabolism 17. As increased plasma lipid levels are associated with an increased risk of atherosclerosis, this may imply that data obtained from isolated, PBMCs could reflect the *in vivo* situation and contribute to biological and clinical insights into the human atherosclerotic process. Changes in the expression of genes related to lipid metabolism and inflammation in PBMCs have been assessed in dietary intervention studies both in large-scale microarray analyses and using single-gene transcripts with quantitative reverse transcription polymerase chain reaction; these data were recently reviewed by de Mello *et al*. 18. Furthermore, gene expression studies in PBMCs seem to reflect changes occurring in metabolically active organs such as the liver, adipose tissue and skeletal muscle 19–21. Therefore, in addition to possibly reflecting changes that occur in metabolically active organs, PBMCs are easily accessible and are also directly involved in the complex inflammation processes that underlie many lifestyle-related diseases.

We have previously reported that intake of FO (1.6 g EPA + DHA) for 7 weeks did not change the levels of serum triglycerides or of markers of inflammation in healthy subjects 22,23, whereas lipidome analyses in the same subjects showed an increased proportion of plasma triglycerides and phospholipids containing long-chain polyunsaturated fatty acids (PUFAs) 24. These results indicate a remodelling of plasma lipids towards a more beneficial profile after intake of FO in healthy subjects. Microarray-based expression profiling quantifying more than 40 000 transcripts was performed to obtain a more comprehensive overview of the processes specifically modulated by EPA and DHA at a molecular level in PBMCs.

## Materials and methods

### Subjects

A total of 36 healthy men and women between 18 and 50 years of age who met the eligibility criteria were included in this study. A detailed description of the protocol, participant recruitment and enrolment, inclusion and exclusion criteria and compliance is given elsewhere 22. In brief, exclusion criteria were fasting levels of total cholesterol >7.5 mmol L^−1^, triglycerides >4 mmol L^−1^, glucose >6.0 mmol L^−1^ and C-reactive protein (CRP) >10 mg L^−1^, body mass index (BMI) ≥30 kg m^−2^ and blood pressure (≥160/100 mmHg). The study was performed at the Akershus University College, Norway between September and December 2009.

### Ethics statement

Written informed consent was obtained from all participants. The study was approved by the Regional Committee of Medical Ethics (approval no. 6.2008.2215) and by the Norwegian Social Science Data Services (approval no. 21924), and was conducted in accordance with the Declaration of Helsinki.

### Study design

**Fig 1:**
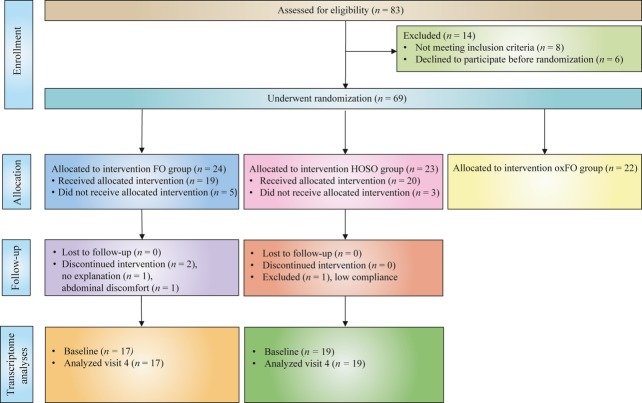
Flow chart showing subjects enrolled, lost during follow-up, and included in the statistical analysis at baseline and after 7 weeks of fish oil supplementation. FO, fish oil; HOSO, high oleic sunflower oil; oxFO, oxidized fish oil. The oxFO group was not included in the present study.

This study was part of a randomized, controlled, double-blind, three-arm, parallel-group study, designed to investigate the health effects of FO intake 22, in which the transcriptome was analysed as a pre-defined endpoint. Subjects were randomly assigned and stratified by gender into three groups using equal randomization (1:1:1). Data from only two of the intervention groups were analysed in the present study (see Fig.1). Subjects were required to take 16 capsules per day containing a total of 8 g day^−1^ of either FO or high oleic sunflower oil (HOSO) for 7 weeks and were instructed to take the capsules with food (during a minimum of two meals). Subjects in the FO group received capsules containing a total of 0.7 g day^−1^ EPA + 0.9 g day^−1^ DHA from cod liver oil (Gadidae sp., EPA/DHA Oil 1200, TINE SA, Oslo, Norway) and subjects in the HOSO group received HOSO purchased from AarhusKarlshamn AB (Malmö, Sweden). The fatty acid composition of the oils has been described elsewhere 22. The capsule containers were identical, and the capsules were the same size and to a large extent similar in colour.

Blood samples were collected for the transcriptome analyses from subjects at visits at 0, 3 and 7 weeks. Prior to the baseline visit (week 0), a 4-week washout period was included in which foods containing marine n-3 fatty acids were excluded from the diet. During the first 3 weeks of the intervention period, a fully controlled isocaloric diet, with all food and beverages provided by Akershus University College, was consumed. The composition of the diet and the food items provided in this study have previously been described 22. During the last 4 weeks of the intervention, the subjects returned to their habitual diet. No intake of fish, fish products, marine n-3-enriched food or additional dietary supplements was allowed during the entire study period of 11 weeks. The study was registered at www.clinicaltrial.gov (identification number NCT01034423).

### Blood sampling

Subjects were asked to refrain from alcohol consumption and vigorous physical activity the day prior to blood sampling. Venous blood samples were collected after an overnight fast (≥12 h) at the same time (±2 h). Serum samples were kept at room temperature for 30 min before centrifugation (1500 ***g*** for 12 min). EDTA tubes with whole blood were kept at room temperature for a maximum of 48 h before counting the total number of lymphocytes and monocytes.

### Routine laboratory analyses

Fasting serum concentrations of high-sensitivity CRP, total-cholesterol, LDL-cholesterol, HDL-cholesterol, triglycerides and glucose as well as lymphocyte and monocyte counts were measured by standard methods in a routine clinical laboratory (Fürst Medical Laboratory, Oslo, Norway).

### PBMCs and RNA isolation

After blood collection, PBMCs were isolated using the BD Vacutainer Cell Preparation tubes according to the manufacturer's instructions (Becton, Dickinson and Co., Franklin Lakes, NJ, USA). Pellets were frozen and stored at −80 °C until further RNA isolation.

Total RNA was isolated from all PBMC samples using RNeasy Mini Kit (Qiagen, Hilden, Germany) with lysis buffer with the addition of β-mercaptoethanol according to the manufacturer's instructions, and stored at −80 °C. RNA quantity and quality were measured using the ND 1000 Spectrophotometer (Saveen Werner AB, Limhamn, Sweden) and Agilent Bioanalyser (Agilent Technologies Inc., Santa Clara, CA, USA), respectively. One sample had an RNA integrity number (RIN) score of 3.3; all other samples were above 8 (mean of 9.6). Nanodrop analysis did not indicate any contamination in the RNA samples.

### Microarray hybridization

Labelled extracts were prepared using Illumina TotalPrep RNA Amplification Kit (Illumina Inc., San Diego, CA, USA) according to the manufacturer's instructions. The labelled extracts were hybridized to an Illumina HumanHT-12 v4 Expression BeadChip and scanned on an Illumina HiScan microarray scanner. Illumina GenomeStudio was used to transform bead-level data to probe-level intensity values and statistics, which were exported raw (unfiltered and non-normalized) for bioinformatic analysis. After hybridization and scanning, a manual quality control step was performed, investigating density plots and hierarchical clustering of raw probe densities. All samples, including the single sample with an RIN score of <8, displayed good characteristics and were included in further analyses.

### Microarray data analyses

Quantile normalization of the Illumina intensities was performed. To improve statistical power, probes without a detectable expression (detection *P*-value >0.01) in at least 10% of the samples were excluded from further analysis. Changes in gene expression were obtained by calculating log2 ratios between the intensities at baseline and after 7 weeks, and the two intervention groups were compared with regard to this ratio. Differentially expressed genes in each group and between groups were identified using the Linear Models for Microarray Data (Limma) 25 package from Bioconductor (http://www.bioconductor.org) with R software. Genes with a nominal *P*-value <0.05 were defined as differentially regulated and subjected to further gene ontology analyses with DAVID software version 6.7 (http://david.abcc.ncifcrf.gov). The list of differentially regulated genes was compared with a reference list (Homo Sapiens), and biological processes containing more than 10 genes and a False discovery rate (FDR) *q*-value of <0.1 was considered significantly modulated.

Gene Set Enrichment Analysis (GSEA) (http://www.broad.mit.edu/gsea/) was applied for the genes defined as expressed in PBMCs. The gene sets in the collection of C2 chemical and genetic perturbations (cgp) and C3 transcription factors from the Molecular Signatures Database (MSigDB) were used separately. Permutation (1000) was performed on phenotypes, and gene sets were defined as significantly changed for FDR *q*-values of <0.25 as recommended for exploratory analyses by Broad Inst.

The leading edge genes contributing to the significance of the regulated gene sets were further analysed for functional and biological pathways using MetaCore (GeneGo, division of Thomson Reuters, St. Joseph, MI, USA) for better interpretation of the GSEA result. MetaCore software is a knowledge database suitable for pathway analysis of experimental data and gene lists. The Minimum information about a microarray experiment (MIAME) standards 26 were followed in the analysis and storage of data. The raw data are available from the Gene Expression Omnibus (GEO) (accession number GSE48368).

### Other statistical analyses

Randomization was carried out by LINK Medical Research AS (Oslo, Norway) using Microsoft Excel and its random generator. Differences in baseline characteristics and number of monocytes and lymphocytes between the groups at baseline or after 7 weeks were compared by Student's *t*-test or Mann–Whitney *U* test when normally and not normally distributed, respectively, using spss for windows (SPSS, version 19.0, IBM Corp, Armonk, NY, USA). The significance level was set to 5% (two-sided).

## Results

### Subject characteristics

A total of 36 normal-weight healthy subjects (26 women and 10 men) completed the study. Subjects were young and middle-aged adults (28.0 ± 8.1 years), with serum lipids within the normal range as shown in Table1. No differences in age, BMI, serum lipids, glucose or total numbers of monocytes and lymphocytes were observed between the FO group (*n *=* *17) and the HOSO group (*n *=* *19) at baseline (Table1) or after 7 weeks of intervention (data not shown). The plasma level of n-3 fatty acids was increased in subjects consuming FO compared to the control group, as previously described 22.

**Table 1:** Baseline characteristics

	HOSO (*n *=* *19)	FO (*n *=* *17)	*P*-value
Male/female	5/14	5/12	
Age (years)	28.6 ± 9.1	27.2 ± 6.9	0.98
TC (mmol L^−1^)	4.9 ± 0.8	4.6 ± 0.8	0.27
LDL-C (mmol L^−1^)	2.7 ± 0.6	2.5 ± 0.8	0.41
HDL-C (mmol L^−1^)	1.5 ± 0.4	1.5 ± 0.3	0.86
TG (mmol L^−1^)	1.0 ± 0.5	0.9 ± 0.6	0.43
Glucose (mmol L^−1^)	4.8 ± 0.5	4.6 ± 0.3	0.21
BMI (kg m^−2^)	23.5 ± 3.1	22.1 ± 2.5	0.16
Monocytes (×10^9^ L^−1^)	0.53 ± 0.17	0.46 ± 0.13	0.20
Lymphocytes (×10^9^ L^−1^)	2.17 ± 0.50	2.11 ± 0.57	0.77

TC, total cholesterol; LDL-C, LDL-cholesterol; HDL-C, HDL-cholesterol; TG, triglycerides; BMI, body mass index.

Data are given as mean ± SD. Differences between the groups were calculated using *t*-test when normally distributed and Mann–Whitney *U* test when not normally distributed (age, triglycerides).

### Gene expression profiling in PBMCs

Microarray hybridization was performed on RNA from PBMCs collected at baseline and after 7 weeks of supplementation from all subjects in both groups. All the microarrays fulfilled the employed quality criteria (see Materials and methods). From the 48 000 probes presented on the HumanHT-12 v4 microarray, 21 236 were defined as expressed in PBMCs.

Differences in gene expression between the two groups were determined by a moderated *t*-test (Limma), by comparing the relative change from baseline to 7 weeks of intervention among the 21 236 expressed genes. Overall, 470 genes were found to be differentially expressed between the two intervention groups (*P *<* *0.05). However, no genes were significantly differentially expressed (FDR *q*-value of <0.1) between the two groups after adjusting for multiple testing (Table S1). The magnitude of the gene expression changes in the FO group ranged between +58% and −23% compared to the control group. Gene transcripts differentially expressed and changed by more than 20% in the FO group compared with the HOSO group are included Table2.

**Table 2:** Gene transcripts differentially expressed and changed by more than 20% in the FO group compared with the HOSO group after 7 weeks of intervention (identified using Limma analyses; *P *>* *0.05)

Gene Symbole	Probe_id	FO vs. HOSO *P*-value	FO vs. HOSO FC
LOC100130100	ILMN_1739508	0.028	1.59
LOC100130100	ILMN_1699214	0.024	1.59
NA	ILMN_1680274	0.017	1.55
NA	ILMN_3240375	0.028	1.54
IGLL1	ILMN_2393765	0.015	1.50
IL32	ILMN_2368530	0.010	1.25
IRF4	ILMN_1754507	0.013	1.22
CCL3L3	ILMN_2105573	0.002	1.20
SIGLEC14	ILMN_3243061	0.045	0.77

NA, not assigned; FC, fold change; FO, fish oil; HOSO, high oleic sunflower oil.

To identify differences across the two intervention groups, we analysed functional relationships among the 470 differentially expressed genes obtained with Limma using the pathway tool DAVID. This tool tests whether any biological processes are significantly enriched among the list of differentially regulated gene transcripts compared to a reference list (Homo Sapiens). Using the list of differentially expressed genes, 236 upregulated and 234 downregulated gene transcripts were identified compared to the HOSO group. The lists of upregulated and downregulated gene transcripts were analysed for enriched biological processes separately. Several biological processes were significantly enriched among the upregulated gene transcripts. These processes are involved in cell cycle and DNA packing and chromosome organization (see Table3). No processes were significantly associated with the genes transcripts that were downregulated by FO compared to HOSO.

**Table 3:** Enriched biological processes in the FO group compared to the HOSO group after 7 weeks of intervention among the upregulated genes obtained with Limma analysis (*P *<* *0.05)

Biological process	Genes	Fold enrichment	*P*-value	FDR
GO:0022402/cell cycle process	KIFC1, MND1, UBE2C, DDIT3, CDK2, CCNE1, MLL5, TUBB, BUB1, MAPRE2, H2AFX, GFI1, HELLS, UBA52	2.63	0.00	0.04
GO:0006323/DNA packaging	H2AFV, H2AFX, NAP1L3, NCAPH2, ASF1B, HELLS	5.62	0.00	0.07
GO:0022403/cell cycle phase	CCNE1, KIFC1, TUBB, BUB1, MND1, H2AFX, MAPRE2, GFI1, UBE2C, HELLS, CDK2	2.83	0.01	0.08
GO:0051276/chromosome organization	KIFC1, MLL5, H2AFV, PRMT7, INO80, H2AFX, NAP1L3, IRF4, NCAPH2, TERF2IP, ASF1B, HELLS	2.64	0.01	0.09
GO:0007049/cell cycle	KIFC1, JAG2, MND1, UBE2C, MCM3, DDIT3, CDK2, CCNE1, MLL5, TUBB, BUB1, MAPRE2, H2AFX, GFI1, UBA52, HELLS	2.19	0.01	0.09

GO, Gene ontology term; FDR, false discovery rate.

Enriched biological processes were identified using DAVID (FDR *q*-value of <0.1).

A moderated *t*-test (using Limma) identified 11 significantly changed gene transcripts in the FO group among the 21 236 expressed genes (FDR *q*-value of <0.1) after 7 weeks. No gene transcripts were found to be significantly changed from baseline after intake of HOSO. Three small nucleolar RNAs (SNORD13, SNORA12 and SNORD12C), two mRNA transcripts coding for VNN2 and the mRNA transcript for CD55 were downregulated after intake of FO for 7 weeks (Table S2).

### GSEA

**Fig 2:**
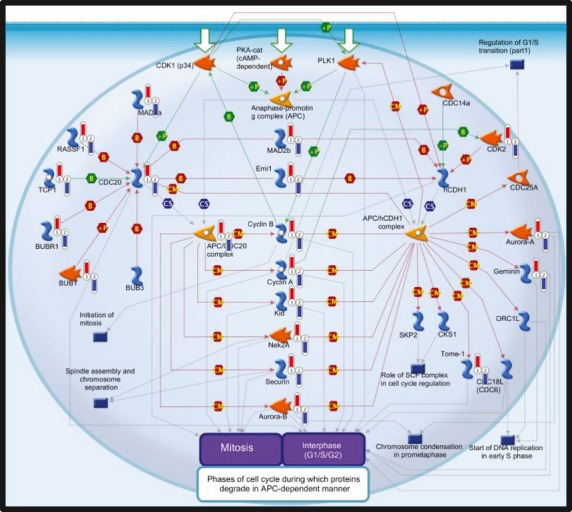
Metacore analysis of leading edge genes. The pathway map showing the role of APC in cell cycle regulation, identified with the Metacore (GeneGo) pathway tool, among the leading edge genes from gene set enrichment analyses using the gene set collection C2 cgp. The leading edge genes were found to be upregulated after intake of fish oil (FO) compared with high oleic sunflower oil (HOSO). The experimental data are shown on the maps as ‘thermometer-like’ figures. Upward thermometers (red) indicate upregulated gene transcripts in the FO group and downward thermometers (blue) indicate downregulated expression levels of the genes in the HOSO group. Further explanations are provided at http://pathwaymaps.com/pdf/MC_legend.pdf. cgp, chemical and genetic perturbations. APC, anaphase-promoting complex.

**Fig 3:**
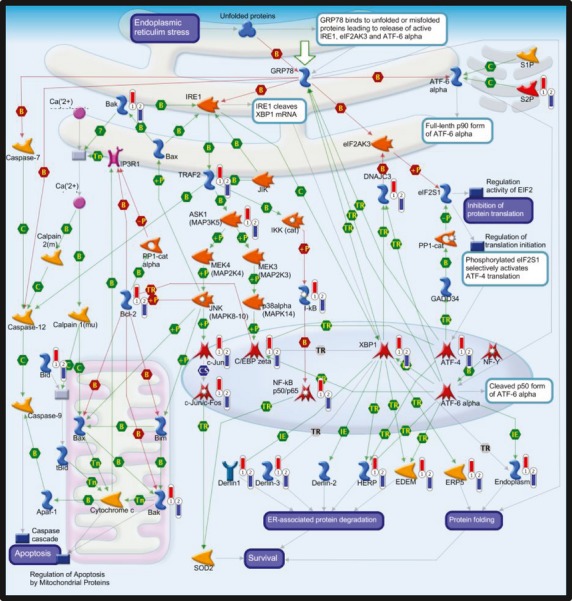
Metacore analysis of leading edge genes. The pathway map: apoptosis and endoplasmic reticulum stress, identified with the Metacore (GeneGo) pathway tool among the leading edge genes from gene set enrichment analyses using the gene set collection C2 cgp. The leading edge genes were found to be upregulated after intake of fish oil (FO) compared with high oleic sunflower oil (HOSO). The experimental data are shown on the maps as ‘thermometer-like’ figures. Upward thermometers (red) indicate upregulated gene transcripts in the FO group and downward thermometers (blue) indicate the downregulated expression levels of the genes in the HOSO group. Further explanations are provided at http://pathwaymaps.com/pdf/MC_legend.pdf.

Gene Set Enrichment Analysis was used to test whether groups of genes involved in the same biological process or pathway, or regulated by the same transcription factor, were changed after intake of FO compared with the control group among the 21 236 defined expressed genes. The relative changes from baseline after 7 weeks of intervention were compared in the two groups. Overall, 162 gene sets were significantly enriched (FDR *q*-value of <0.25; 72 with FDR *q*-value of <0.15) in the FO group compared with the HOSO group in the C2 cgp collection (MSigDB). These gene sets included several overlapping genes. Leading edge genes (1460 non-overlapping genes shown in Table S3) contributing to the significance of the regulated gene sets were further analysed for functional and biological pathways using MetaCore for better interpretation of the GSEA result. The results were ranked by their *P*-value and annotated onto the developed pathway maps. It is interesting that several pathways were significantly regulated among the leading edge genes. The top 10 pathway maps (FDR *q*-value of <0.05) were related to cell cycle, apoptosis, immune response, protein folding and maturation, and DNA damage (Table4). Metacore pathway maps of the Anaphase-promoting complex (APC) regulation of cell cycle and endoplasmic reticulum (ER) stress response are shown in Figs2 and 3, respectively.

**Table 4:** Leading edge genes identified with the C2 cgp collection (see Table S4) are visualized using Metacore

Pathway maps	Ratio
Cell cycle. The metaphase checkpoint	36/21
Protein folding and maturation. POMC processing	30/18
Immune response. IL-2 activation and signalling pathway	49/22
Cell cycle. Role of APC in cell cycle regulation	32/17
Immune response. CD16 signalling in NK cells	69/24
Cell cycle. Start of DNA replication in early S phase	32/16
Cell cycle. Spindle assembly and chromosome separation	33/15
Apoptosis and survival. Endoplasmic reticulum stress response pathway	53/19
DNA damage. ATM/ATR regulation of G1/S checkpoint	32/14
Apoptosis and survival. FAS signalling cascades	44/16

FDR, false discovery rate; ATM, ataxia-telangiectasia mutated; ATR, ATM- and rad3-related; APC, anaphase-promoting complex; NK, natural killer; FAS, tumour necrosis factor receptor superfamily member 6.

The top 10 pathways are shown (FDR *q*-value of <0.05). Ratio indicates the number of genes represented in the pathway maps/number of genes identified in the leading edge analyses.

Finally, to explore the regulatory mechanisms underlying the change in gene regulation we performed a GSEA analysis to test whether groups of genes with common *cis*-motifs were differentially regulated among the defined expressed genes. Forty-nine gene sets were significantly upregulated after intake of FO, compared with HOSO, using the C3 collection (MSigDB) (Table S4). These included genes regulated by a common *cis*-acting element with binding sites for 35 known transcription factors such as E2F transcription factor, nuclear respiratory factor 1 (NRF1), tumour protein 53 (TP53), SP1 transcription factor (SP1), activating transcription factor and 2 (ATF4 and ATF2), Forkhead box O4 (FOXO4), macrophage migration inhibitory factor (MIF), signal transducer and activator of transcription 1 (STAT1), hypoxia inducible factor 1, alpha subunit (HIF1A), sterol regulatory element binding transcription factor 1 (SREBP1) and hepatic nuclear factor 4 alpha (HNF4A) (Table5).

**Table 5:** Gene set enrichment analyses. Enriched gene sets from the C3 transcription factor targets collection (MSigDB) in the FO group compared to the HOSO group

Gene set	NES	*P*-value	Transcription factor
V$SP1_Q2_01	1.63	0.00	SP1 transcription factor
V$E2F_Q6_01	1.61	0.01	E2F: TFDP1
V$HNF4_01	1.59	0.00	HNF4A, NR2A1: hepatic nuclear factor 4 alpha
V$PAX8_01	1.57	0.03	PAX 8 (paired box 8)
V$TBP_01	1.57	0.01	TBP (TATA box binding protein)
V$CREBP1_Q2	1.56	0.00	ATF2 (activating transcription factor 2)
V$PAX5_01	1.55	0.01	PAX5 (paired box 5)
V$TATA_C	1.54	0.01	TAF8: TBP-associated factor
V$LMO2COM_01	1.54	0.00	LMO2: LIM domain only 2 (rhombotin-like 1)
V$NRF1_Q6	1.51	0.00	NRF1 (nuclear respiratory factor 1)
V$SP1_01	1.50	0.02	SP1 transcription factor
V$FOXO4_01	1.49	0.02	FOXO4 (forkhead box O4)
V$ATF4_Q2	1.49	0.02	ATF4 (activating transcription factor 4)
V$COUP_01	1.49	0.02	NR2F1
V$AP4_Q6_01	1.48	0.01	AP4 (AP4 zinc finger protein)
V$MIF1_01	1.48	0.04	MIF: macrophage migration inhibitory factor (glycosylation-inhibiting factor)
CTAWWWATA_V$RSRFC4_Q2	1.47	0.01	MEF2A (myocyte enhancer factor 2A)
V$GRE_C	1.47	0.01	NR3C1: nuclear receptor subfamily 3, group C, member 1 (glucocorticoid receptor)
V$SREBP_Q3	1.46	0.01	SREBF1 (sterol regulatory element binding transcription factor 1)
V$STAT1_03	1.45	0.02	STAT1 (signal transducer and activator of transcription 1)
V$HIF1_Q3	1.45	0.01	HIF1a (hypoxia-inducible factor 1, alpha subunit)
V$AP4_Q5	1.44	0.02	AP4 (AP4 zinc finger protein)
V$RP58_01	1.44	0.02	ZBTB18 (zinc finger and BTB domain containing 18)
V$ZIC1_01	1.43	0.03	ZIC1 (Zic family member 1)
V$COREBINDINGFACTOR_Q6	1.43	0.02	CBFA2T2 (core-binding factor, runt domain, alpha subunit 2; translocated to, 2)
V$AP1_Q6_01	1.43	0.03	JUN (Jun oncogene)
V$CP2_01	1.42	0.03	TFCP2 (transcription factor CP2)
V$SREBP1_Q6	1.42	0.04	SREBF1 (sterol regulatory element binding transcription factor 1)
V$EVI1_02	1.42	0.03	EVI1 (ecotropic viral integration site)
V$TAL1BETAITF2_01	1.42	0.01	TAL1 (T-cell acute lymphocytic leukaemia 1)
V$P53_02	1.42	0.04	TP53 (tumour protein 53)
V$STAT_Q6	1.40	0.04	STAT1 (signal transducer and activator of transcription 1)
V$FOXJ2_01	1.40	0.03	FOXJ2 (forkhead box J2)
V$MYCMAX_B	1.39	0.02	MAX (MYC associated factor X)
V$ZIC2_01	1.39	0.03	ZIC2 (zic family member 2)

NES, normalized enrichment score; FO, fish oil; HOSO, high oleic sunflower oil; MSigDB, Molecular Signatures Database.

Gene sets with *cis*-acting binding sites for known transcription factors are presented. Gene sets with an FDR *q*-value of <0.25 were considered significantly enriched in the FO group.

Transcriptome analyses were also performed after 3 weeks of intervention. The gene transcripts identified as differentially expressed according to Limma analyses (*P *<* *0.05) were involved in the cell cycle, chromosome organization, response to oxidative stress, translation and regulation of apoptosis (data not shown). Using GSEA analyses, no gene sets were identified as differentially expressed between the two groups after 3 weeks.

## Discussion

We have investigated the transcriptome profile of PBMCs after intake of FO in a randomized controlled dietary intervention trial with healthy subjects. Intake of FO containing 1.6 g day^−1^ DHA and EPA for 7 weeks modulated gene transcripts related to biological processes and pathways such as cell cycle, ER stress and apoptosis compared with control (intake of HOSO). These processes and pathways were identified using an exploratory approach with several data analysis strategies. A similar transcriptome profile was observed after 3 weeks of intervention (results not shown); however, the effect was more prominent at the end of the 7-week intervention.

Gene transcripts involved in several phases of the cell cycle were identified as significantly modulated after intake of FO compared with control (intake of HOSO). Modulation of cell cycle gene transcripts was characterized by increased expression of cyclins and cyclin-dependent kinases as shown in Fig.2 and Table3. Cyclins and cyclin-dependent kinases are involved in and control the transition through G1, S, G2 and the M phase of cell cycling 27. Our results are in accordance with previously published data demonstrating upregulation of several key gene transcripts (CDK2, CDK4, CCND2, PCNA, MCM6) involved in cell cycle and DNA synthesis in PBMCs after intake of FO (1.8 g day^−1^ EPA + DHA) in elderly subjects for 26 weeks 28. In addition, pathways related to the cell cycle were upregulated by FO and slightly downregulated after intake of HOSO 28,29. Taken together, intake of FO may increase key regulatory genes involved in cell cycle progression at a transcriptional level in PBMCs. n-3 fatty acids have previously been shown to inhibit cell proliferation and cell growth of cancer cell lines *in vitro* 30–32. Key cell cycle regulators and target proteins of cancer therapy were recently found to be downregulated by DHA in a human colon cancer cell line *in vitro* 31,33. The discrepancies observed between the effect of n-3 fatty acids on cancer cells *in vitro* and in studies of PBMCs from healthy subjects may be related to the fact that cancer cells have lost normal cell functions and are characterized by mutations in genes controlling cell cycle, repair functions and/or cell death.

We did not observe any change in the total number of monocytes or lymphocytes after intake of FO, indicating that the potential activation of cell cycle progression is accompanied by induction of cell death. This is supported by the observation that gene transcripts involved in the ER stress response and apoptosis were upregulated after intake of FO compared with control (HOSO). ER stress is induced under conditions of cellular stress and activates the unfolded protein response (UPR) to maintain cellular homeostasis 34, and sustained ER stress may lead to apoptosis 35. ER stress is associated with several diseases such as cancer, neurodegenerative disorders and atherosclerosis 36–38. In this study, we found that genes with the common regulatory motifs for ATF4 and MIF1 were upregulated after intake of FO. ATF4 is a transcription factor involved in ER stress and the UPR response 34, and MIF1 was recently linked to the degradation of proteins during the UPR response 39. In agreement with our finding, the gene transcript of MIF1 in PBMCs was found to be significantly increased after intake of FO in the elderly 28. Recent observations in cancer cell lines have shown that DHA may induce ER stress and increase ATF4 both at the protein and transcriptional levels 31. How n-3 fatty acids induce ER stress is not known. However, the n-3 fatty acids EPA and DHA are susceptible to oxidation and may form lipid peroxidation products that could influence the cellular redox state, lead to oxidative stress and thereby induce ER stress. It is interesting that a higher expression level of autophagy-related genes was recently identified in individuals with a high plasma ratio of n-3/n-6 compared to those with lower ratios 40. Autophagy is a prosurvival mechanism that may have a role in restoring cellular homeostasis after oxidative and ER stress 41 and is also closely linked to lipid metabolism 42.

Increased expression of genes that share common regulatory elements for the transcription factors E2F, TP53, STAT1, FOXO4 and SP1 further highlights the fact that intake of FO modulates cell cycle and apoptosis. In accordance with our findings showing increased expression of TP53 target genes, the mRNA transcript of TP53 were found to be elevated by Bouwens *et al*. 28 following intake of FO in PBMCs.

The expression of target genes with the regulatory motifs for NRF1was also increased after FO supplementation when compared with the HOSO group. NRF1 target genes are involved in mitochondrial function and biogenesis. Impairment of mitochondrial functions is associated with age-related disorders such as type 2 diabetes and Alzheimer's disease 43,44, and increased mitochondrial number has been associated with the life-extending effects of exercise and energy restriction in rats 45,46. Similar to our findings, intake of a diet rich in antioxidants and PUFAs has been shown to regulate NRF2 target genes in blood cells of healthy subjects 47. We have also identified increased expression of genes with the regulatory motif for HIF1A after intake of FO. HIF1A controls the hypoxic response occurring at low oxygen tension and HIF1A target genes are involved in stress and defence responses 48. Of note, Bøhn *et al*. 47 also found that HIF1A target genes were induced at a transcriptional level in blood cells. Taken together, these findings suggest that changes in gene transcripts towards an optimization of defence and stress processes may represent a potential way to maintain cellular functions upon exposure of redox active components in food.

Changes in the whole-genome expression profile in PBMCs or blood cells after intake of FO or n-3 supplements in healthy subjects, the elderly or patients with dyslipidaemia or Alzheimer's disease have been reported in several studies, and the changes are associated with lipid metabolism, antioxidant defence and inflammation 28,49–53. However, the biological significance of these previous studies should be determined with caution as either no control was included 52, the mRNA samples from the subjects were pooled 49–51 or the within-group changes in the intervention group and the control group were reported separately 28,53.

The present study has several strengths including the double-blind, randomized, controlled study design and the use of several data analysis strategies in which the effect of FO intake was compared with that of HOSO. Among the limitations are the relatively short intervention period and the small number of subjects included. The genes identified as responsive to intake of FO were not significantly differentially expressed following adjustment for multiple testing. This is in agreement with the findings of other nutrigenomic studies and is probably related to the small effect of nutrients on gene expression, both with regard to the proportion of gene transcripts changed and to the induction of the changes in gene expression concordance with large inter-individual variations 29,47. The magnitude of the gene expression changes observed ranged between +58% and −23% compared with the control group and is in line with the findings of other dietary transcriptome studies 28,53. However, these moderate changes over time may still have an impact on health and lifestyle-related disease 54. In the current exploratory study, we therefore focused on regulated biological processes and groups of genes (gene sets) rather than single genes to identify the effect of FO on the transcriptome profile. The processes and pathways identified were upregulated in the FO group compared with the HOSO group. Whether these effects are specific effects of n-3 fatty acids rather than other long chain PUFAs such as n-6 PUFAs remains to be elucidated.

To our knowledge, this is the first study to identify changes in gene expression after intake of FO compared with a control group in a dietary intervention study. Using an exploratory approach, we have identified enriched biological processes and pathways related to cell cycle, ER stress response and apoptosis after intake of FO. These processes and pathways are involved in normal cell function and may ultimately influence whole body health.

## Funding

This study was supported by the Research Council of Norway (project no. 184813/110), Oslo and Akershus University College of Applied Sciences, Norway, the University of Oslo, Norway and the Throne Holst Foundation for Nutrition Research.

## Authors' contributions

MCWM, SMU, IO, GIB, KWB and KBH were responsible for study concept and design; MCWM, SMU, IO, ER and KBH conducted the research; MCWM, SMU, C-CG, IO, MH, ER, MT and KBH performed data analysis and interpretation; MCWM, SMU, IO and KBH wrote the manuscript; and all authors approved the final version to be published.

## Conflict of interests statement

KWB is a clinical nutritionist/project manager at the TINE SA R&D Center, Norway, with no financial interest. None of the other authors has any conflict of interests.

## Abbreviations

FO: fish oil
HOSO: high oleic sunflower oil
PBMCs: peripheral blood mononuclear cells
EPA: eicosapentaenoic acid
DHA: docosahexaenoic acid
Limma: Linear Models for Microarray Data
GSEA: gene set enrichment analyses

## Supporting Information

Table S1 Genes differentially expressed between the two goups (*P* < 0.05).

Table S2 Significantly regulated gene transcripts (FDR < 10%) after intake of fish oil for 7 weeks compared to baseline. The fold change from baseline is presented. Gene transcripts were identified with paired moderate *t*-test (Limma). The corresponding values in the HOSO group are presented.

Table S3 Leading edge genes contributing to the significance of the enriched gene sets in the FO group.

Table S4 GSEA analyses. Enriched gene sets in the FO group compared with the HOSO group from the C3 transcription factor collection (MSigDB). Gene sets with FDR *q*-value <0.25 were consider significantly enriched in the FO group.
