# Supplementary material for: Fish oil supplementation induces expression of genes related to cell cycle, endoplasmic reticulum stress and apoptosis in peripheral blood mononuclear cells: a transcriptomic approach
Source: J Intern Med. 2014 Mar 20;276(5):498–511. doi: 10.1111/joim.12217 (PMC4263263; doi:10.1111/joim.12217)
Supplement: Table S2 — Significantly regulated gene transcripts (FDR < 10%) after intake of fish oil for 7 weeks compared to baseline. The fold change from baseline is presented. Gene transcripts were identified with paired moderate t-test (Limma). The corresponding values in the HOSO group are presented. [file joim0276-0498-SD2.docx]

|  | **FO** | | **HOSO** | |
| --- | --- | --- | --- | --- |
| **Gene** | **FDR q-value** | **FC** | **FDR q-value** | **FC** |
| SNORD13 | 0.01 | 0.60 | 0.26 | 0.69 |
| VNN2 | 0.01 | 0.80 | 0.67 | 0.96 |
| CD55 | 0.01 | 0.85 | 0.86 | 0.98 |
| RSBN1L | 0.03 | 0.88 | 0.26 | 0.91 |
| SNORA12 | 0.03 | 0.70 | 0.38 | 0.86 |
| VNN2 | 0.04 | 0.77 | 0.57 | 0.93 |
| LOC80054 | 0.09 | 1.09 | 0.26 | 1.08 |
| RAB11FIP2 | 0.09 | 0.90 | 0.39 | 0.95 |
| SERPINB9 | 0.09 | 0.85 | 0.69 | 0.97 |
| POLR1D | 0.09 | 0.90 | 0.83 | 0.98 |
| SNORD12C | 0.1 | 0.88 | 0.48 | 0.95 |

**Supplemental Table S2.** Significantly regulated gene transcripts (FDR < 10%) after intake of fish oil for seven weeks compared to baseline. The fold change from baseline is presented. Gene transcripts were identified with paired moderate t-test (Limma). The corresponding values in the HOSO group are presented

.FO: Fish oil, HOSO: High oleic sunflower oil, FC: Fold change
